# Modulation of free energy landscapes as a strategy for the design of antimicrobial peptides

**DOI:** 10.1007/s10867-022-09605-z

**Published:** 2022-04-14

**Authors:** Sergio A. Hassan, Peter J. Steinbach

**Affiliations:** Bioinformatics and Computational Biosciences Branch, National Institute of Allergy and Infectious Diseases, National Institutes of Health, Bethesda, MD 20892 USA

**Keywords:** Antimicrobial peptides, Peptide structure prediction, Molecular dynamics, Implicit solvent model, Drug design, Machine learning

## Abstract

Computational design of antimicrobial peptides (AMPs) is a promising area of research for developing novel agents against drug-resistant bacteria. AMPs are present naturally in many organisms, from bacteria to humans, a time-tested mechanism that makes them attractive as effective antibiotics. Depending on the environment, AMPs can exhibit α-helical or β-sheet conformations, a mix of both, or lack secondary structure; they can be linear or cyclic. Prediction of their structures is challenging but critical for rational design. Promising AMP leads can be developed using essentially two approaches: traditional modeling of the physicochemical mechanisms that determine peptide behavior in aqueous and membrane environments and knowledge-based, e.g., machine learning (ML) techniques, that exploit ever-growing AMP databases. Here, we explore the conformational landscapes of two recently ML-designed AMPs, characterize the dependence of these landscapes on the medium conditions, and identify features in peptide and membrane landscapes that mediate protein-membrane association. For both peptides, we observe greater conformational diversity in an aqueous solvent than in a less polar solvent, and one peptide is seen to alter its conformation more dramatically than the other upon the change of solvent. Our results support the view that structural rearrangement in response to environmental changes is central to the mechanism of membrane-structure disruption by linear peptides. We expect that the design of AMPs by ML will benefit from the incorporation of peptide conformational substates as quantified here with molecular simulations.

**Supplementary information:**

The online version contains supplementary material available at 10.1007/s10867-022-09605-z.

## Introduction

A growing number of infections are becoming increasingly difficult to treat because the causative pathogens can mutate and become resistant to existing antibiotics [1–3]. This race against pathogenic bacteria is ongoing, as new drugs can become ineffective in a few years. Some strains have already acquired resistance to all or nearly all known antibiotics, including those considered as last resort [4]. The few options left in the therapeutic arsenal have sparked concerns that the “antibiotic era” may be nearing its end [5]. New approaches are needed to design effective and potent drugs. Antimicrobial peptides (AMPs) [6–8] exhibit strong antibacterial properties, as measured, e.g., by the minimal concentration (MIC) needed to inhibit bacterial growth. AMPs are present naturally in many organisms [8, 9], from bacteria, where they act against other bacterial species in their competition for resources, to humans, as a first line of defense of the immune system. This ubiquitous antimicrobial mechanism has sustained hopes that short peptides, if appropriately designed, could form the basis of effective and potent antibiotics, either of broad utility or for a specific species (e.g., *E. coli*, *S. aureus*), strain, or group (gram-positive or gram-negative). However, as is often the case in drug development, poor understanding of their mechanisms of action, i.e., the specific molecular processes underlying the suppression of bacterial growth, has hampered progress. Several mechanisms are known, and others have been proposed [7, 10, 11]. AMPs can act on a variety of targets in bacterial cells. Some mechanisms of action involve modulation of the host immune system, which is redirected to kill and clear the foreign pathogen. Others include internal (cytosolic) targets, such as enzymes necessary for cell survival and growth, or membrane-bound proteins and complexes, such as lipid receptors, e.g., for lipid II, which is needed for the synthesis of the protective peptidoglycan wall, the complex polymeric mesh-like structure that separates the inner and outer lipid membranes [12, 13].

Of particular interest are mechanisms that disrupt the inner membrane, mechanisms for which bacteria may have fewer options to develop resistance, as that would require a reconfiguration of the lipid bilayer. The precise molecular mechanisms of the disruption of membrane structure are system dependent and typically unknown. Much effort has been devoted to elucidating them in specific cases, and some models have been proposed [7, 10, 11]. Cell death results either from the formation of transmembrane pores leading to leakage of ions or from more extensive damage to the membrane integrity, whereby water, ions, ATP, and other small and large (DNA/RNA) molecules can escape the cell interior. The result is either cell depolarization or lysis.

The computational design of an AMP entails optimizing the peptide sequence to bind and disrupt the bacterial inner membrane while leaving the mammalian membrane intact. AMP development is challenging because therapeutic value also depends on toxicity, proteolytic degradation, and oral bioavailability. Organism selectivity is possible because bacterial and mammalian cell membranes differ in composition. The inner membrane of bacterial cells is typically anionic, whereas the mammalian cell is zwitterionic. They are also composed of a mixture of lipid types (e.g., POPG, cardiolipin in bacteria; POPC, cholesterol in mammalian cells) that differ in their head-group chemistries, lengths of hydrocarbon chains, and relative concentrations. Their interactions with a particular peptide depend on its amino acid composition and on the conformations it adopts in the aqueous phase.

Given the complexity and size of these systems, the elucidation of membrane-structure disruption by molecular simulation alone is a daunting challenge. Therefore, a data-science approach may prove to be valuable, as some of the techniques devised, such as machine learning (ML), can extract valuable, non-obvious information from available data [14, 15]. ML sidesteps molecular details, instead modeling correlations in various input data, e.g., peptide amino acid sequence and MIC [16]. The price of this simplification is that large amounts of data are needed for training before attempting reliable predictions. These training data must ultimately be obtained from experiments or computer simulations. If successful, the algorithm could predict whether a given sequence, not previously seen by the machine, would exhibit antimicrobial activity. If adequately trained, it might predict whether the peptide is selective for a specific bacterial species, strain, or group, or whether it would be toxic to mammalian cells. Several repositories of AMP data have been created, with the DBAASP being one of the largest and most comprehensive databases available [17].

Recent de novo design of AMPs produced short peptides exhibiting high antimicrobial activity and low hemolytic activity [16]. Two of these peptides, denoted SP4 and SP15, were suggested to affect the permeability of bacterial membranes differently. Both enhance permeability at high concentrations (~ 100 $\mathrm\mu$g/mL), but at lower concentrations closer to each peptide’s MIC, SP4 and SP15 were observed to behave differently. At 12.5 $\mathrm\mu$g/mL SP4 affected permeability; at 3.125 $\mathrm\mu$g/mL SP15 did not.

We have simulated the conformations and dynamics of the SP4 and SP15 peptides in aqueous and membrane-like environments at 37 ºC to help interpret their effects on membrane permeability. For each peptide-solvent pair, we have modeled the energy landscape [18] at two levels of approximation. We performed structure-prediction calculations, in which an efficiently calculated, continuum representation of each solvent was used to facilitate conformational exploration sufficient to make reasonable predictions of the predominant conformations sampled at equilibrium. The stabilities of these predicted conformational families were then assessed using a computationally more intensive, all-atom description of each solvent. Finally, we performed Monte Carlo simulations to show how peptide binding modes differ among membranes and conformational families and to obtain initial peptide-membrane configurations for elucidating molecular mechanisms through dynamics simulation.

Our results suggest that the ability of a peptide to change its conformation in response to environmental changes, along with the existence of multiple conformational substates in equilibrium, may be central to the experimental observations. The significance of the connection between a biomolecule’s functionally important motions and its environment has been appreciated for some time, thanks in large part to the pioneering work of Hans Frauenfelder. With his spectroscopic studies of heme proteins, Frauenfelder illuminated the protein energy landscape [18], revealing a large number of conformational substates [19] arranged in a hierarchy [20], even for a relatively simple protein like myoglobin. By exploring ligand rebinding and protein dynamics over wide ranges of temperature [19, 21], pressure [22], viscosity [19, 23, 24], and pH [25], his lab characterized quantitatively both the thermodynamics of the protein-solvent system at equilibrium [26], i.e., the relative populations of conformational substates, and the kinetics of transitions among the substates [21].

After discussing our simulation results, future avenues of study are outlined. We anticipate that properties of the peptide-solvent system such as those highlighted here, and others computed from statistical ensembles in physics-based simulations, will improve our ability to design AMPs by machine learning.

## Methods

The peptide sequences are GIKFFLKKLKKHI (SP4) and RWIRWVWRKKLR (SP15), with charged N- and amidated C- termini and all residues in the L- configuration. Both peptides are highly cationic, as K^+^ and R^+^ were protonated and H was assumed to be deprotonated in both the aqueous and non-aqueous environments.

In the SCPISM, the electrostatic contribution to the effective energy of the system (here, a peptide immersed in a solvent *s*) is described by two terms, a sum over interatomic interactions and a sum over atomic self-energies, in the form [27, 28]

1 $${E}_{elec}^{s}=\frac{1}{2}\sum_{i\ne j}^{\mathcal{N},\mathcal{N}}\frac{{q}_{i}{q}_{j}}{{r}_{ij}{D}_{s,ij}({r}_{ij};\mathbf{r})}+\frac{1}{2}\sum_{i=1}^{\mathcal{N}}\frac{{q}_{i}^{2}}{{R}_{s,i}\left(\mathbf{r}\right)}\left\{\frac{1}{{D}_{s,i}\left[{R}_{s,i}\left(\mathbf{r}\right);\mathbf{r}\right]}-1\right\}$$

where $\mathcal{N}$ is the number of atoms, ${r}_{ij}$ is the distance between atoms *i* and *j*, and ${q}_{i}$ and ${R}_{i}$ are the charge and effective radius of atom *i*. The function $D$(*x*; **r**) is sigmoidal in *x*, and **r** denotes the dependence of $D$ on the conformation of the system. This dependence is introduced through a parameter α, which determines the rate of increase of $D$ with *x*. From a physical standpoint, α contains information on the volume, polarity, and polarizability of the liquid molecules and depends on external control parameters, such as temperature [27]. If *x* is the distance *r* from a central charge $q$, then $D$ is given by [29]

2 $$D\left(r;\alpha \right)=\frac{1+{\varepsilon }_{s}}{1+k{e}^{-\alpha r}}-1$$

where *k* ≡ (ε_*s*_ − 1)/2. So, $D$ increases from unity at small distances to the bulk dielectric constant at large distances. For short peptides, the reduction in screening due to solvent exclusion by neighboring solute atoms is small [27, 28]. Here, the parameter quantifying an exponential distance dependence of this effect [28] was taken to be 20 Å for both the interaction and self-energy terms.

An atom’s effective radius in the solvated protein is taken to be the weighted average of its radius in bulk water, ${R}_{w,i}$, and its radius in a bulk protein environment, ${R}_{p,i}$, i.e., ${R}_{s,i}={\xi }_{i}{R}_{w,i}+\left(1-{\xi }_{i}\right){R}_{p,i}$, where ${\xi }_{i}$ is the fractional solvent-accessible surface area (SASA) of the atom [27, 30]. The nonpolar effects of solvation (cavity formation) are approximated for an aqueous solvent as ${G}_{np}=a+{\sum }_{i}{\sigma }_{i}SAS{A}_{i}$, where $a$ and $\sigma$ are positive parameters. Seeking the simplest description of the continuum solvent, the same atomic surface tension, 5.2 cal/mol/Å^2^, was used here for all atoms to estimate the nonpolar free energy of solvation, and the approximation ${G}_{np}\sim 0$ was used for octanol. Parameters used in approximating $SAS{A}_{i}$ [31] were taken from the same or a similar atom type in the CHARMM22 force field without modification. The additional $\psi$ torsion angle employed previously [31] to modify the protein force field was omitted here.

The total energy in the SCPISM is thus ${E}_{SCP}^{s}={E}_{elec}^{s}+{G}_{np}$, with empirical adjustments to account for the effects of liquid-structure forces on H-bond interactions [28, 32]. A correction to the Lennard-Jones term has also been introduced to implicitly account for the effects of the solvent dispersion forces [33]. The SCPISM is parameterized based on experimental hydration energies of amino acid side-chain analogs [27] and results from MD simulations of the potentials of mean force between amino acid side-chain interactions in water [28]. For the structure calculations performed in this paper, the model was simplified by removing the H-bond and van der Waals corrections and reparametrized based on structure-prediction calculations of the trp-zip 2 [34] and trp-cage [35] peptides with the CHARMM36 force field [36, 37], as described [31].

In the derivation of Eq. 1, the energy is zero for the system composed of the preformed solvent and all the protein atoms in the vacuum, at infinite separation from one another and the solvent [27, 38]. Therefore, it is possible to obtain the vacuum-to-water transfer energy (hydration) $\Delta {E}_{h}={E}_{SCP}^{w}-{E}_{SCP}^{0}$ and the water-to-octanol transfer energy $\Delta {E}_{ow}={E}_{SCP}^{o}-{E}_{SCP}^{w}$ =  − *RT* log *P*, where *P* is the partition coefficient. Both quantities can be calculated from a peptide conformational ensemble, or for a single structure, as obtained with ${E}_{SCP}^{w}$ and ${E}_{SCP}^{o}$ in each solvent.

In general, the charge of the peptide can change due to changes in the pH of the environment or from local pKa shifts of titratable groups at a fixed pH due to conformational changes. Charges can be calculated self-consistently in the context of the SCPISM using a variational approach [39, 40]. Here, all charges were held constant.

Replica-exchange [41, 42] Langevin dynamic simulations were performed, as described previously [31], using 24 replicas for trp-zip 2 and trp-cage (during parameterization) and 22 replicas for SP4 and SP15. Temperatures ranged from the experimentally relevant temperature to about 1470 K. Replicas were simulated at very high temperatures to promote the crossing of energy barriers separating conformational substates; the analysis was performed at the lowest (experimental) temperature. Simulations included 996,000 replica-exchange steps, each involving 100 steps of Langevin dynamics followed by swaps being considered between adjacent temperatures. A collision frequency of 2 ps^−1^ was used for all non-hydrogen atoms. Electrostatic interactions were shifted to zero at 10 Å, and Lennard–Jones interactions were switched off from 8 to 10 Å [43]. The SHAKE algorithm was used for bonds involving hydrogen atoms. The integration time step was 1.5 fs for temperatures below 700 K and 1.0 fs for higher temperatures. The first and last residues were excluded when calculating root-mean-square deviation (rmsd).

Nine screening parameters in the SCPISM, the α values in Eq. 2, were varied between a minimum value of 0.1 Å^−1^ and a maximum of 7.0 Å^−1^ to simultaneously favor native-like conformations of the peptides trp-zip 2 and trp-cage. As before [31], low energy conformations sampled in simulations initiated from both extended and NMR-determined conformations were collected for different parameterizations and used to bias the parameters iteratively. Atoms were again classified as being part of a charged group (q), a backbone hydrogen-bonding group (b), or any other neutral group (n). For electrostatic interactions, the combination rule sometimes used, α_ij_ = (α_i_ α_j_)^1/2^, was relaxed. The iterative refinement resulted in the following three self-energy α values (in Å^−1^), α_q_ = 0.1, α_b_ = 6.23, α_n_ = 6.36, and six interaction-energy values, α_qq_ = 6.98, α_bb_ = 0.48, α_nn_ = 0.55, α_qb_ = 5.24, α_qn_ = 1.57, α_bn_ = 6.59. The folding simulations of trp-zip 2 and trp-cage performed using these refined parameters are depicted in the Supplementary Information, Fig. S1. These parameters were then used to simulate SP4 and SP15 in water. To approximate an octanol environment, all nine screening parameters were set to 0.1 Å^−1^.

Atomistic dynamics simulations were performed to assess the stability of the structures predicted with the new SCPISM parameterization in the corresponding solvents. The simulations were carried out in the NPT ensemble, at 37 ºC and 1 atm, in a cubic cell with periodic boundary conditions ( PBC) and particle-mesh Ewald (PME) summation, using the all-atom representation of the CHARMM36 force field. Bonds to hydrogen atoms were constrained with the SHAKE algorithm, and a 2-fs time-step was used to integrate the equations of motion. The pressure was maintained with the Langevin piston method; the mass and collision frequency were set at 400 amu and 20 ps^−1^. The temperature was maintained with the Hoover thermostat, using a mass of 10^3^ kcal mol^−1^ps^2^. The length of the cube’s sides was initially set at ∼9.3 nm and filled with ~ 27,000 TIP3P water or 2400 1-octanol molecules, yielding average densities of ∼0.993 g∕cm^3^ and ∼0.815 g∕cm^3^, respectively, after equilibration. Six or seven Cl^−^ were added to neutralize SP4 and SP15, respectively, in both water and octanol, whereas 74 Na^+^ and 74 Cl^−^ ions were used for the ~ 150-mM NaCl aqueous solutions. All the ions were randomly distributed in the solvent phase after the peptides were solvated and the overlapping solvent molecules removed. An initial 4-ns dynamic phase was performed while keeping the peptide atoms fixed to let the solvent molecules and ions adjust to the peptide’s electric field. A snapshot at the end of this phase was taken as the initial configuration for the subsequent simulations of the corresponding systems. The constraints were then removed, and after heating and equilibration, 15-ns MD simulations were conducted for each peptide. The last 12 ns were analyzed.

Two anionic (POPA, POPG) and two zwitterionic (POPC, POPE) phospholipid bilayers were built as models for bacterial and mammalian cell membranes. Each membrane consisted initially of a square slab with a side length of 12 nm and a width of 5 nm, containing 324 lipid molecules per leaflet, with surfaces parallel to the (x, y) plane and centered at z = 0. The membranes were then immersed in a cubic box with a side length of 12 nm filled with TIP3P water molecules, and potassium ions were added to neutralize the total charge of POPA and POPG. PME summations were used and tetragonal (PBC) were applied so that the (x, y) and z dimensions were free to adjust independently; a harmonic restraint with a force constant of 0.1 kcal mol^–1^ Å^–2^ was applied in the (x, y) plane to prevent the lipids’ heavy atoms from moving outside the [–6.25, + 6.25] nm range in each direction. All other simulation parameters were as described above for the peptide simulations. After heating and equilibration at 35 ºC and 1 atm, the simulations were extended for 4 ns to obtain the structures used in the peptide-membrane multiscaling simulations. Simulated-annealing MC simulations [44] were performed for each peptide conformation in the presence of each membrane. The peptides were treated as rigid bodies; so, the conformational ensemble obtained at the lowest temperature (here, 35 ºC) represents first-encounter modes, i.e., the likely distribution before any relaxation occurs. We used the CHARMM force field for the peptides and membranes, and the original SCP model [27, 32] was used to account for the solvent effects. The structure of the peptides and the corresponding effects of the solvent were adapted on the fly using a multiscaling algorithm [45]. The temperature was lowered in ten steps through a logarithmic schedule staring at 726 ºC; 2 × 10^5^ trial moves were sampled per temperature, each consisting of either translation (one-third of the moves; restricted to < 2-nm center-of-mass displacements), rotation (one-third of the moves; restricted to < 180º around randomly chosen axes through the peptide’s center of mass), or roto-translation (one-third; < 2 nm and < 180º) taken from homogeneous distributions. Simulations were performed inside a rigid sphere of 11-nm diameter centered on the membrane to minimize artifacts introduced by the lipids at the slab’s edges; no corrections were introduced to the a priori probability due to the sphere potential.

## Results

**Fig. 1:**
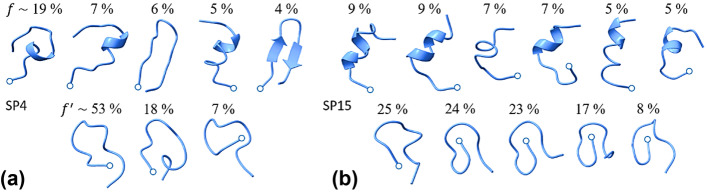
Main conformational families of SP4 (**a**) and SP15 (**b**) in water (upper row) and octanol (lower), as predicted at 37 ºC from replica-exchange simulations, using all-atom representations of the peptides and continuum descriptions of the solvents. $f$ and $f{^{\prime}}$ represent the populations of the corresponding conformational families in water and octanol. Both peptides are ~ 50% disordered in water, whereas only ~ 20% and < 5% are disordered in octanol. Circles indicate N-termini

For SP4 and SP15, the first 96,000 replica-exchange steps simulated were ignored, and 9000 structures were saved for analysis, one every 100 steps, and clustered as described previously [31]. The clustering reflects the conformations sampled appreciably for the two AMPs simulated in the two continuum solvents (Supplementary Information, Figs. S2-S5). The simulations produced several conformational substates, or families, in water and octanol; each family is represented by the cluster’s centroid, or hub, as shown in Fig. 1. Both peptides are more disordered in water, with ~ 50% of the population unstructured (i.e., many families, each with small population) and the rest adopting local secondary structure, mainly helical. In contrast, only 20% of the population of SP4 is unstructured in octanol; the disorder of SP15 is negligible. Note that different families may have similar physicochemical characteristics, as clusters can be somewhat similar, e.g., clusters 3 and 5 for SP4 in water (Fig. S2). The enhanced mobility of both peptides in the aqueous environment, approximated here as a continuum, is consistent with the increased fluctuations seen in explicit-water simulations of other small proteins, e.g., myoglobin [46, 47].

None of the structures in octanol shows traces of secondary structure, and each seems to favor a cyclic-like conformation where the N-terminus (charged in these models) plays a role in stabilizing the hubs. It is important to note that the SCPISM parameters for octanol have not been optimized; so, these results are tentative and intended to illustrate. However, the absence of helical structure in octanol is not unreasonable since such a fold would expose the charged side chains, which are distributed along the entire sequence, to the low-permittivity solvent, which is energetically unfavorable. Although the OH group of octanol could hydrogen-bond to the side chains, reducing the electrostatic penalty, such interactions would be absent inside a membrane.

**Fig. 2:**
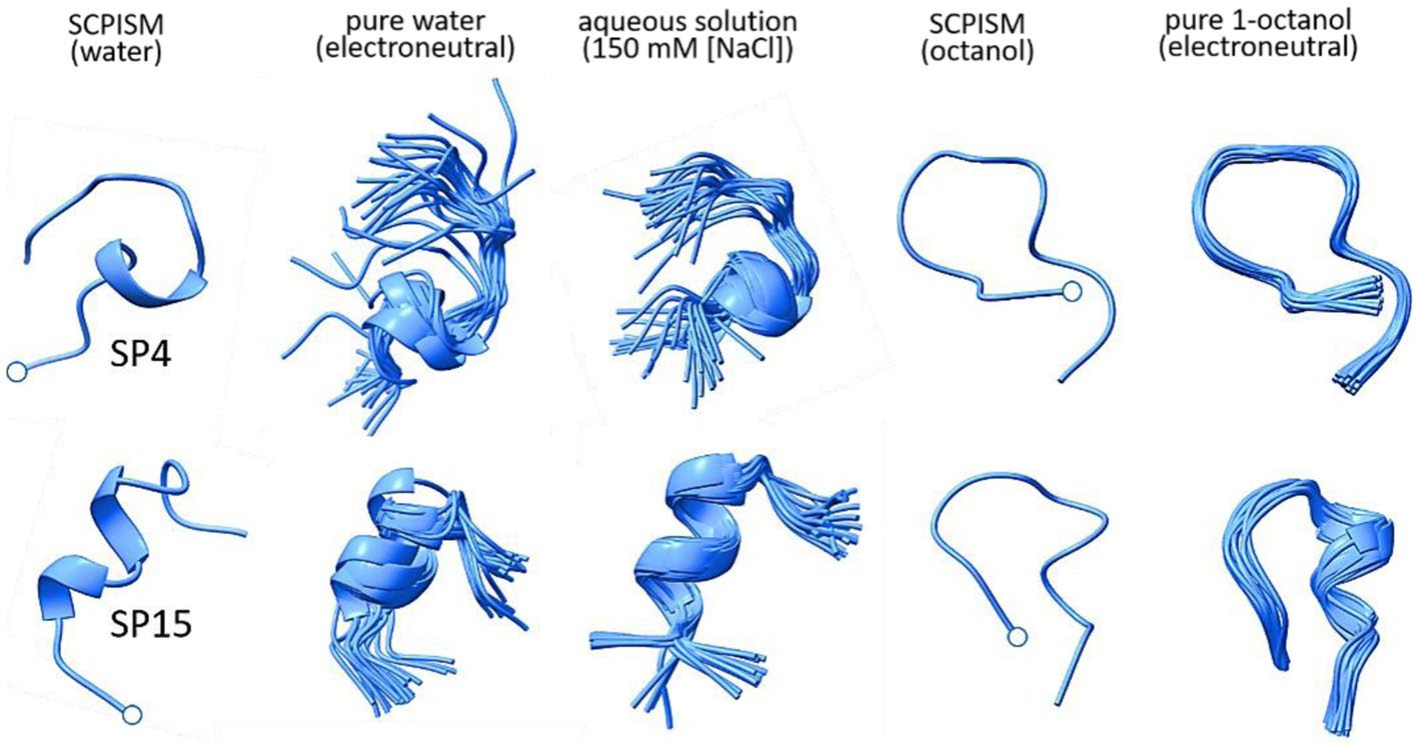
Snapshots from 15-ns molecular dynamics simulations of the conformations with the highest population in Fig. 1. The simulations were performed at 37 ºC and 1 atm, with all-atom representations of the peptides and solvents. Circles indicate N-termini

Molecular dynamic simulations were carried out in explicit solvents to probe the structural stability of the predicted conformations (cf. Methods). The main hub in water was solvated both in pure water (electrically neutral) and in an aqueous solution (150 mM of NaCl); the main hub in octanol was immersed in pure 1-octanol (electroneutral). The structures remained stable, undergoing the expected fluctuations about the predicted fold (Fig. 2). Ions can participate in stabilizing some of the side chains, both in solution and in electrically neutral water. Despite the lower permittivity of octanol, which promotes side-chain–backbone H-bonds due to stronger electrostatics, the OH groups compete for H-bonding as well, forcing some of the side chains in octanol to become solvent exposed during the dynamics (not shown), a situation less likely to occur in a lipid membrane, unless the peptide forms H-bonds with the head groups. Although gratifying, these observations do not guarantee that the structures are at a global free-energy minimum, as they might be kinetically trapped, especially in the much more viscous 1-octanol.

**Fig. 3:**
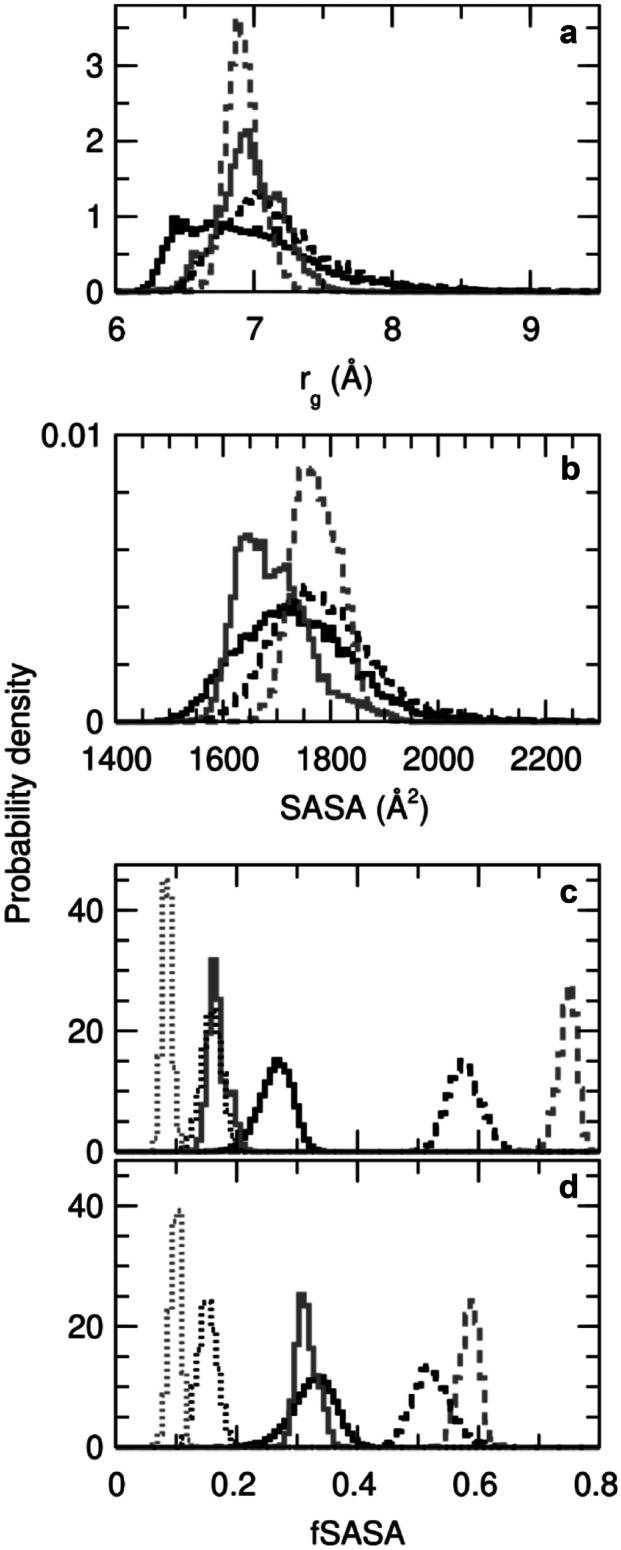
Normalized histograms calculated from simulations of SP4 and SP15, initiated from extended conformations, in water-like (black) and octanol-like (gray) continuum solvents. Results are plotted for the radius of gyration (**a**), solvent-accessible surface area (**b**), and the fraction of solvent-accessible surface area of atoms in different groups for SP4 (**c**) and SP15 (**d**). In a and b, SP4 results are solid and SP15 results are dashed. In c and d, results for atoms in positively charged, hydrophobic, and hydrophilic groups are solid, dashed, and dotted, respectively

The 9000 structures analyzed for each of the four continuum-solvent simulations were characterized by the peptide’s radius of gyration (Fig. 3a) and total solvent accessible surface area (Fig. 3b). Both quantities show a broader range of structures was sampled in the aqueous environment than in the less polar solvent, consistent with a long history of experiments and explicit-water simulations that show protein hydration promotes protein motion. In particular, SP4 in an aqueous solvent was seen to adopt especially small values of the radius of gyration (Fig. 3a, solid black line), even though SP4 is one residue longer than SP15. Similarly, the changes in surface exposure of charged, hydrophilic, and hydrophobic groups upon the change in environment are all greater for SP4 (Fig. 3c) than for SP15 (Fig. 3d). The simulations show that SP4 is intrinsically more flexible than SP15 and, thus, better able to adapt structurally to changes in the polarity of its environment. This greater sensitivity to environment might account for the observation that SP4 disrupts the structure of bacterial membranes at concentrations close to its MIC, unlike SP15.

## Discussion

Recent computational studies have led to a series of short (< 15 residues) AMPs, some with MICs consistent with potent antibiotics [16]. The basis for these studies has been an ML algorithm trained with a few hundred known AMPs obtained from the DBAASP [17]. The input data consisted of a series of physicochemical features of the peptides: hydrophobic moment, hydrophobicity, charge density, isoelectric point, membrane-penetration depth, angle with respect to the membrane surface, propensity for disorder, and propensity to aggregate. These properties may indeed determine a peptide’s potential to perturb the membrane structure. However, the formal calculation of these quantities requires a priori knowledge of the peptide structure. Unfortunately, the structures of most AMPs are not known; so, the authors assumed that all the AMPs adopted α-helical conformations. This assumption was used during training and inverse design. The method produced a set of AMPs with low MIC and high therapeutic index.

The structure of a peptide depends on the medium in which it is immersed, both the solvent composition (e.g., water vs. lipid) and conditions, e.g., pH and salt concentration, all of which change as a peptide migrates between environments. While temperature and pressure dramatically change the free energy landscape, the focus here is on physiological values. Peptides are generally flexible in solution, coexisting in multiple conformational families with interconverting populations. A given family may select a particular protein or bind preferentially to one site among many in a protein [48, 49]; likewise, a family may recognize and associate preferentially to a particular membrane. Experimental detection and characterization of peptide conformations in aqueous media are challenging. NMR spectroscopy can produce one (most common) or more distinct conformations with atomic resolution, but assumptions need to be made on their populations and kinetics [50, 51]. The problem is equally challenging in a membrane, but some structural insight can be obtained using oriented CD [52]. Available data show that linear peptides can adopt a variety of conformations, including α-helix, β-sheet, or a mix of both, or be intrinsically disordered or in a random-coil state [11]. Experimental data are extremely valuable when training a machine, and the use of octanol as a general membrane-like medium might provide physically meaningful information while avoiding the experimental challenges posed by real membranes.

The alternative to experiments is prediction through computer simulations. (The “intermediate” solution by homology, commonly used in modeling proteins, or the more recent and accurate ML method based on artificial neural networks [53], are not reliable given the sensitivity of peptide conformations to local changes in the sequence, the scarcity of experimentally resolved structures in solution, and the likely presence of multiple conformers.) However, several conceptual and practical questions arise, mainly on the choice of the environment for which structures are to be predicted. For example, upon approaching and binding to a membrane, the peptide may or may not penetrate it. As the carpet model [10] suggests, peptides could accumulate on the membrane surface and damage its structure through various mechanisms, likely dependent on the peptide sequence and chemistry of the lipid head groups. In such cases, structure prediction in water is more reasonable. On the other hand, the peptide may fully penetrate the membrane, in which case prediction of structures in a lipid environment makes more sense. In general, the peptide penetrates the membrane only partially, in a process governed by a complex interplay of forces. Thus, the problem becomes one of predicting structures of a peptide in the water/membrane system and somehow incorporating physicochemical features from all the structures to train the machine. Computer simulations under these conditions become impractical because multiple systems need to be simulated to have enough training data for the algorithm to learn.

**Fig. 4:**
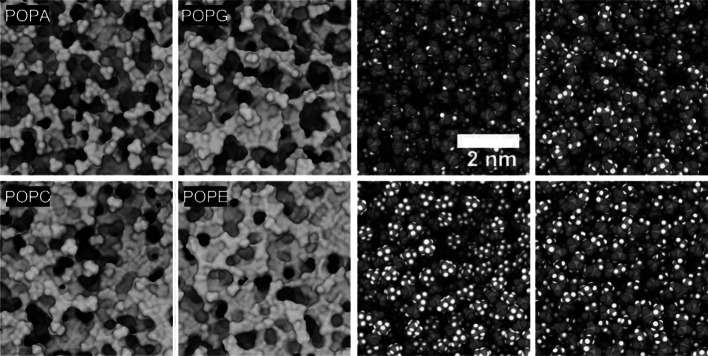
Physicochemical features of bacterial (upper row; POPA and POPG) and mammalian (lower; POPC and POPE) model cell membranes. Left panel: solvent-contact surfaces of equilibrated structures at 35 ºC and 1 atm, showing different degrees of roughness determined by the head groups' interactions with one another and with water and ions (ions not shown; cf. Fig. S6). Right panel: same snapshots, with the surface removed to reveal the atomic arrangement of the head group (van der Waals representation; P: orange, O: red, H: white, C: gray, N: blue). The local surface topography and the atomic composition within the cracks and crevices select the peptide and the particular conformational family that can bind to the membrane

**Fig. 5:**
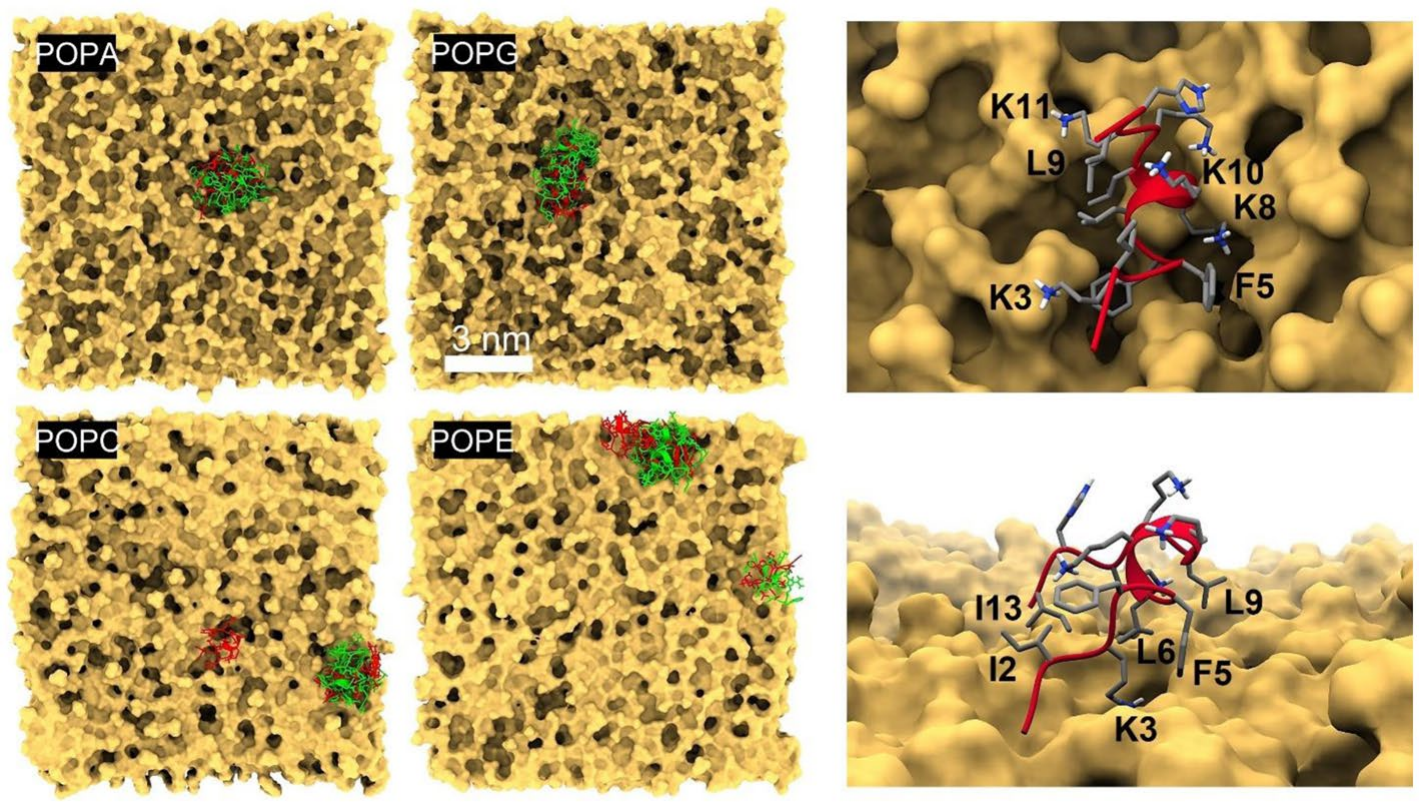
Left: Binding of SP4 (red) and SP15 (green) conformational families obtained from multiscaling simulations at 35 ºC. The representative member of each conformational family was simulated separately, showing that the peptides tend to recognize specific crevices that form on the membrane surface. Right: details of the interactions of SP4, second family (cf. Figure 1a), with POPA (upper) and POPC (lower), showing the binding modes induced by the head groups: K3, K8, K10, and K11 interact strongly with the phosphate groups of POPA, whereas I2, F5, L6, L9, and I13 interact strongly with the methyl groups of POPC

One or more of the structures predicted in water may have the potential to bind either the anionic or zwitterionic membranes, or both, and perturb their structure. This can be seen from several characteristics known to affect biomolecular interactions, binding, and stabilization, such as surface electrostatics, the distribution of polar groups, the ratio of polar/nonpolar surface areas, and the number of donors and acceptors on the peptide that are solvent exposed and ready to engage the lipid head groups, provided the chemical composition of the head groups favors the interaction (Fig. S6 and Fig. 5). It will be important to include such membrane features in training any ML algorithm. First-encounter binding modes between a peptide and a membrane can be calculated by Monte Carlo simulations. These modes can then be used as the starting configurations for subsequent MD simulations to investigate the sequence of events that lead to membrane-structure disruption in a particular peptide/membrane system. To expedite the systematic calculation of lead compounds, a computationally efficient method is critical. A method was proposed previously [45] based on an adaptive multiscaling algorithm that treats the interacting partners at different levels of resolution, speeding up computation significantly. Binding is controlled by the structural granularity of the membrane surfaces (Fig. 4, left panel), akin to binding pockets on protein surfaces, and by the chemical characteristics of the head groups (Fig. 4, right). Their relevance as features in any physics-based ML approach is apparent. Surface granularity and chemical composition are important in protein–ligand interactions, as well, but the membrane surface is much more fluid than a protein, producing a more complex conformational landscape with more substates. The noise introduced by this highly mobile membrane/liquid interface may be enough to trigger conformational changes in an approaching peptide or protein (e.g., through stochastic resonance [54, 55] coupled to one of the peptide’s slow harmonic motions), aside from other, more direct physical effects of highly charged interfaces on the peptide’s structure, kinetics, and dynamics [56]. The membrane can also be very highly charged, which creates a layer of bound counterions (Stern layer) that must be locally disrupted for a cationic peptide to bind (Fig. S6). Figure 5 shows the first-encounter binding modes of the main conformational family of SP4 and SP15 to anionic and zwitterionic membranes. Because the predicted peptide conformations possess similar spatial distributions of charged and hydrophobic moieties, it is not surprising that they tend to recognize similar regions on the membrane surface, suggesting the presence of “weak spots” the peptides might use to penetrate it. The head groups determine the mode of binding, and a given peptide adopts different orientations in the presence of different membranes, as hydrophilic and hydrophobic moieties associate with complementary groups in the membrane. For example, the negatively charged phosphate groups in POPA attract the Lys^+^ and Arg^+^ side chains, while the methyl groups in POPC attract nonpolar residues, flipping the peptide such that the polar/charged residues remain hydrated.

This multiscaling method was used previously to obtain the binding modes and affinities of ultrasmall nanoparticles (protein mimics) for anionic and zwitterionic phospholipid membranes and showed how selectivity for a membrane can be achieved by manipulating the particle design [57]. An ML algorithm was used to optimize the inverse design [58]. Similar ideas can be applied to linear peptides: physicochemical features can first be inferred from desirable properties, e.g., MIC, and amino acid sequences can then be optimized to reproduce the features. Many AMPs have cyclic topology, naturally linked by disulfide bridges. Using the features as the basis for a pharmacophore should allow the design of cyclic AMPs as well.

We intend to build on this work by developing a model to design AMPs using physicochemical peptide features that can be quantified in different environments, e.g., water and octanol, along with those characterizing various membranes. Conformation-dependent peptide features include all those considered previously [16] in the derivation of SP4 and SP15 and others we deem critical, such as polar and nonpolar surface areas, or the number of solvent-exposed donor and acceptor atoms. Membrane features would include the ratio of lipid types (since binary and ternary mixtures are not uncommon), size and charge of functional groups, number of potential proton donors and acceptors, and possibly others.

The calculation of such features is straightforward once the peptide conformations are identified in each solvent. To predict the conformational families for SP4 and SP15, we have used replica-exchange Langevin dynamics and a computationally efficient forcefield, with an atomistic representation of the peptide and a continuum representation of the solvent. The continuum model was proposed previously [27, 28, 30] and used in several applications, including the structure prediction of peptides up to 35-residues long and the dependence of their conformations on the solvent conditions [28, 31, 59]. In the SCPISM, the electrostatic effects are based on elementary notions of dielectrics [27, 29], making apparent the physics involved (e.g., polarity, polarizability, molecular volume, and temperature). It is thus possible to use the model in any solvents for which the assumptions of a continuum make physical sense, e.g., small molecular volumes and fast rotational and translational diffusions; other than water, few solvents qualify. Although these assumptions do not hold for lipids in a membrane, where steric forces due to the oriented hydrocarbon chains can be strong, it is possible to capture implicitly the effects of removing water from the space occupied by the lipids [28]. These water-exclusion effects are critical, strengthening the solute–solute electrostatic interactions, including their contributions to H-bonds. Other essential solvent-mediated effects are hydrophobicity, dispersion forces, and H-bonds, all incorporated empirically; steric effects are typically included through Langevin dynamics.

## Conclusions

Our simulations of two linear antimicrobial peptides, SP4 and SP15, show that for both peptides, the predominant family of structures in octanol is more than twice the size of the largest family in water. The aqueous environment produces an energy landscape more easily explored, enabling transitions among conformational substates. We observed that SP4 undergoes a greater conformational change upon moving from an aqueous environment to one far less polar. The fractional solvent accessible surface areas of charged, hydrophilic, and hydrophobic groups change more dramatically for SP4 than SP15. Assuming that the peptides enter the membrane, this enhanced rearrangement of SP4 may contribute to an increase in the permeability of the bacterial membrane at relatively low concentration, close to its measured MIC. This process may be general for linear AMPs that act through this particular mechanism of action. Clearly, a mechanistic understanding of the structural perturbation of bacterial membranes, but not others, would require a detailed simulation approach. As illustrated here, fully atomistic simulations of peptides in the presence of different lipid bilayers are needed to address this question. Nonetheless, differences in environmental dependences obtained with efficient, continuum treatments of water and octanol phases may be sufficiently realistic to inform and improve predictions by machine learning.

## Supplementary information

Below is the link to the electronic supplementary material.

Supplementary file1 (PDF 1581 KB)
