# Supplementary material for: Modulation of free energy landscapes as a strategy for the design of antimicrobial peptides
Source: J Biol Phys. 2022 Apr 14;48(2):151–66. doi: 10.1007/s10867-022-09605-z (PMC9054992; doi:10.1007/s10867-022-09605-z)
Supplement: Supplementary file 1 — Supplementary file1 (PDF 1581 KB) [file 10867_2022_9605_MOESM1_ESM.pdf]

**Supplementary Information**

# Modulation of Free Energy Landscapes as a Strategy for the Design of Antimicrobial Peptides


Sergio A. Hassan and Peter J. Steinbach

Bioinformatics and Computational Biosciences Branch, National Institute of Allergy and Infectious Diseases, National Institutes of Health, Bethesda, MD 20892, USA

Email: steinbac@mail.nih.gov

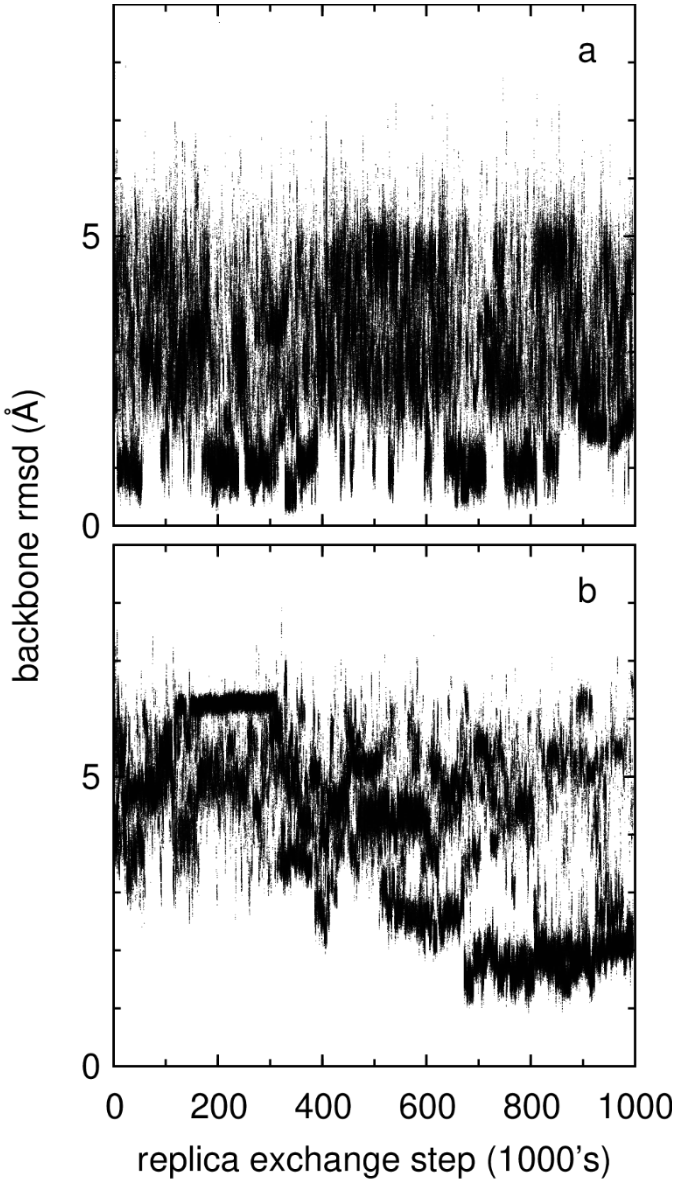


Figure S1. Backbone root mean-squared deviation from the NMR structure for folding simulation initiated from an extended conformation of a) trp-zip 2 at 288 K and b) trp-cage at 282 K.

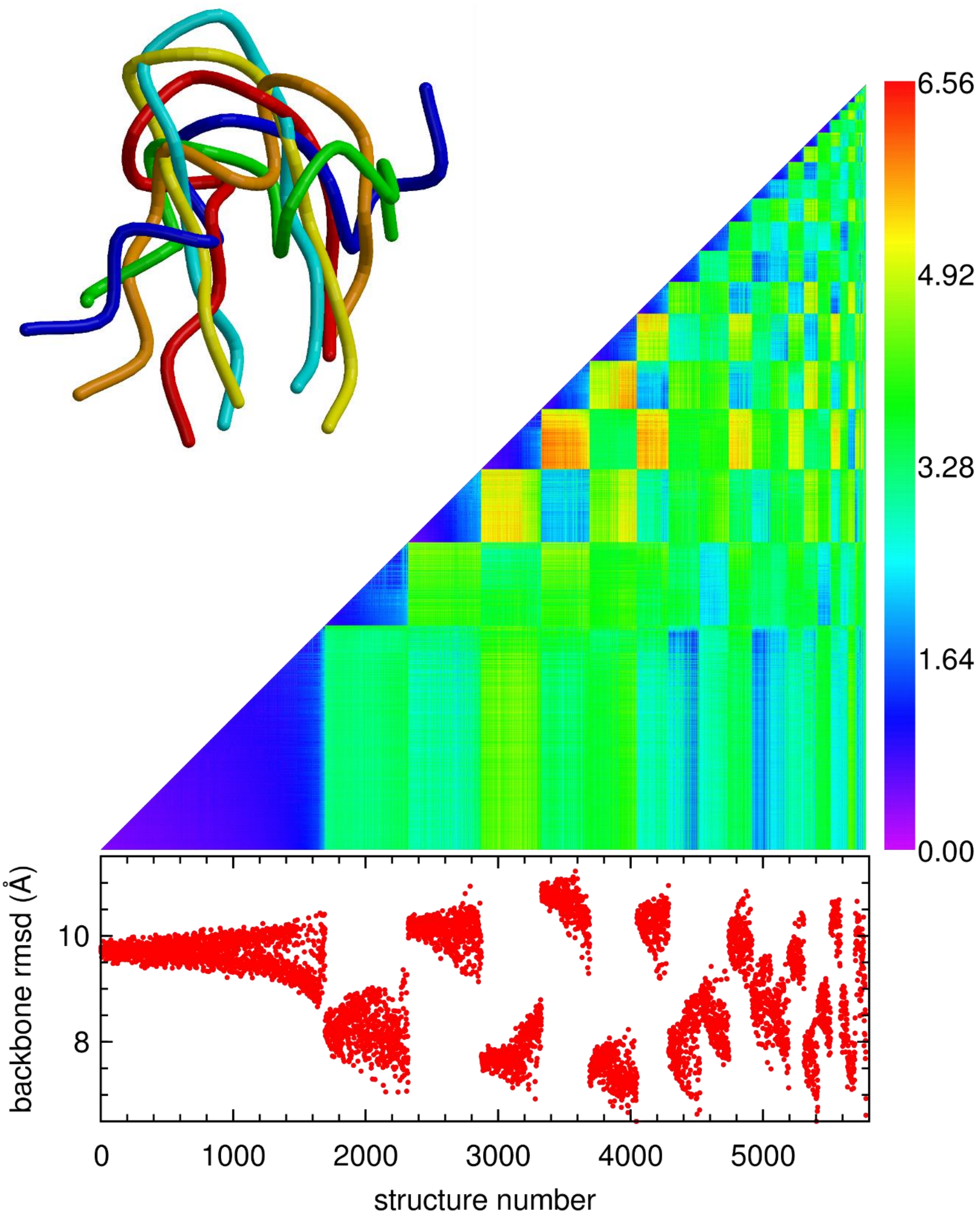


Figure S2. Conformations sampled for SP4 in continuum aqueous solvent. Top: Matrix of rms backbone deviations between structures, after clustering, deleting small clusters, and reclustering. Clusters are ordered from largest to smallest and cluster members by rms deviation from the centroid. Scale at right is in Å. Centroids of clusters are superimposed as α-carbon traces, colored in decreasing order of cluster size (red, orange, yellow, green, cyan, blue).
Bottom: Backbone rms deviation from initial extended structure.

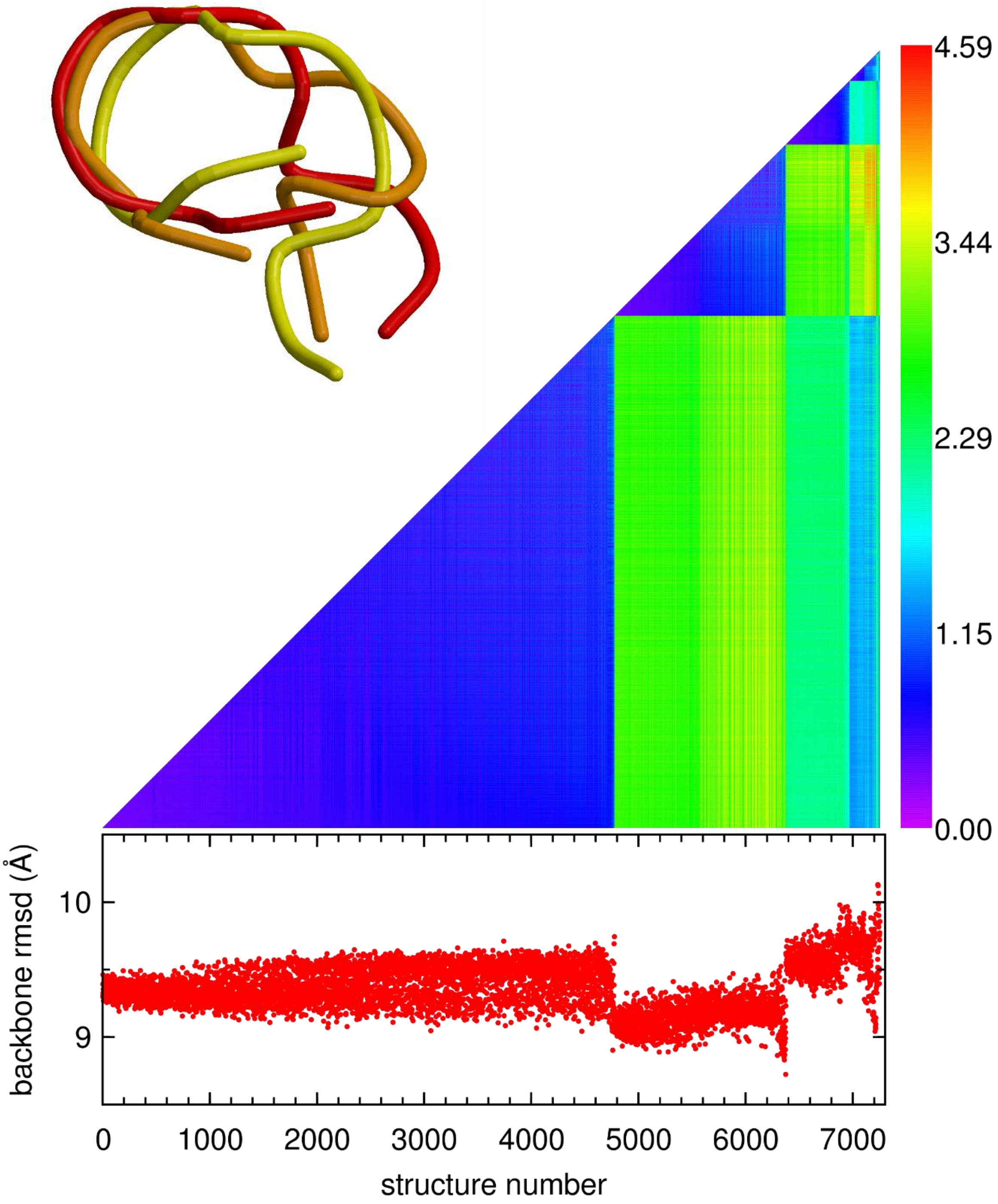


Figure S3. Conformations sampled for SP4 in continuum apolar solvent, depicted as in Figure S2.

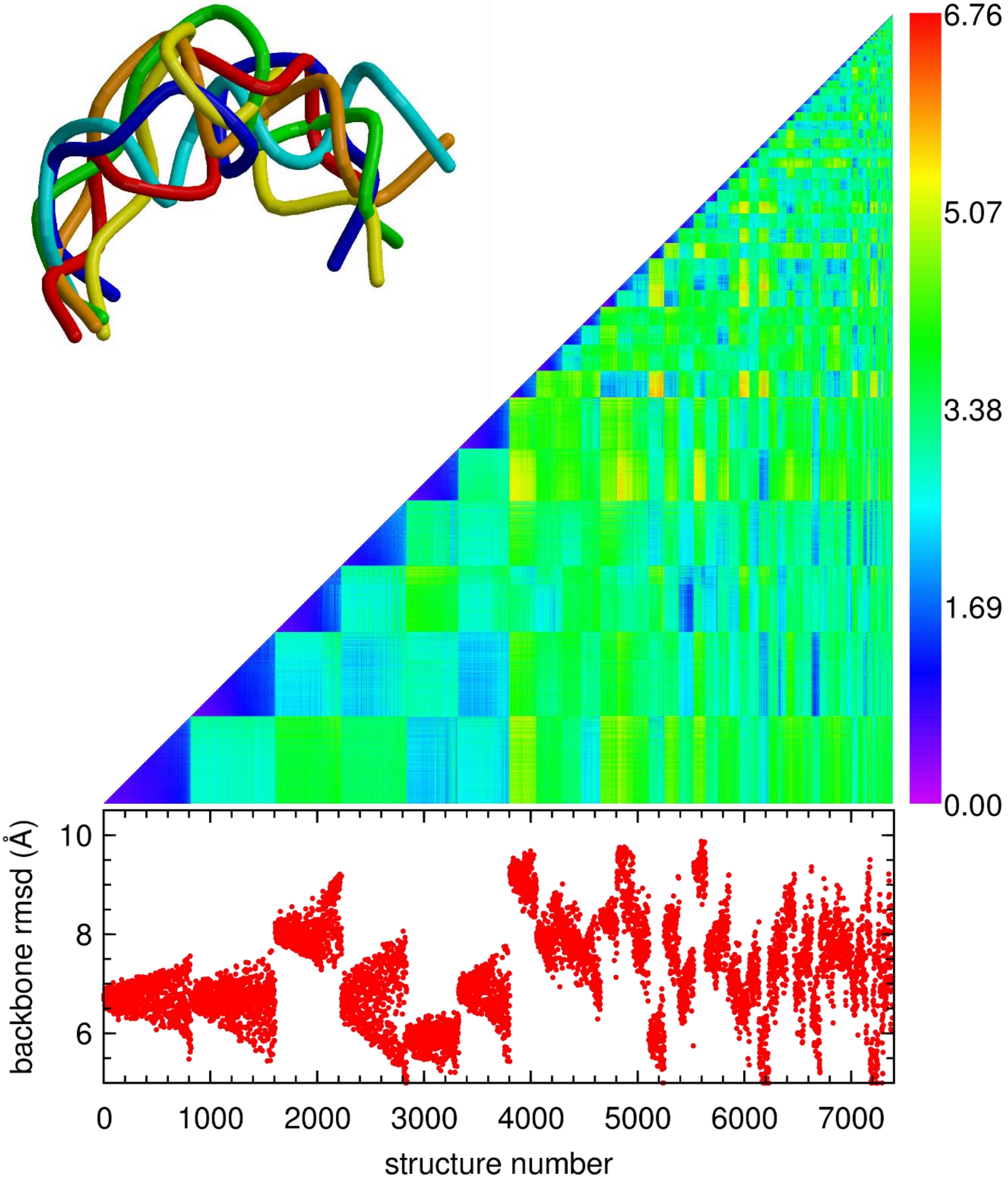


Figure S4. Conformations sampled for SP15 in continuum aqueous solvent, depicted as in Figure S2.

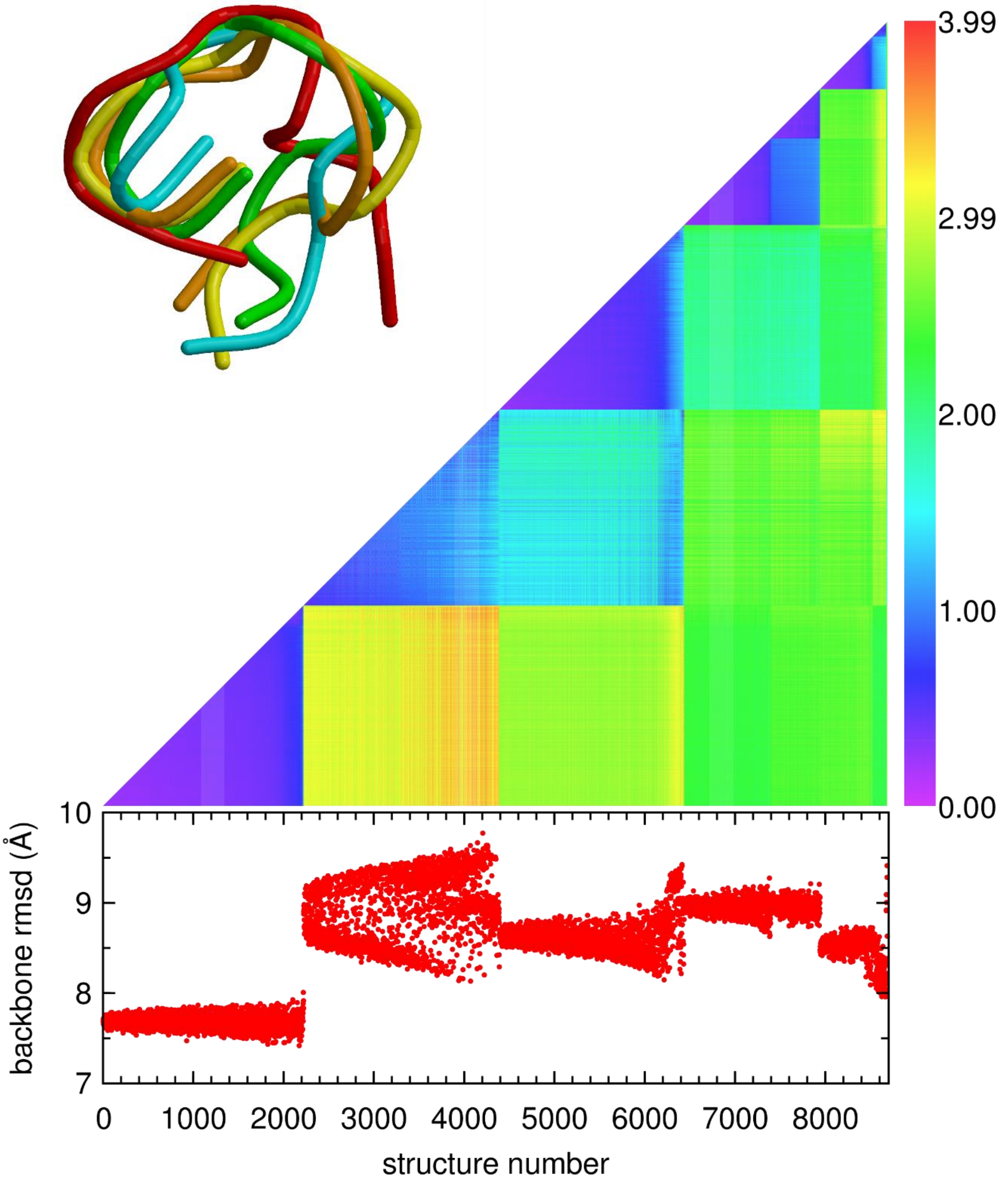


Figure S5. Conformations sampled for SP15 in continuum apolar solvent, depicted as in Figure S2.

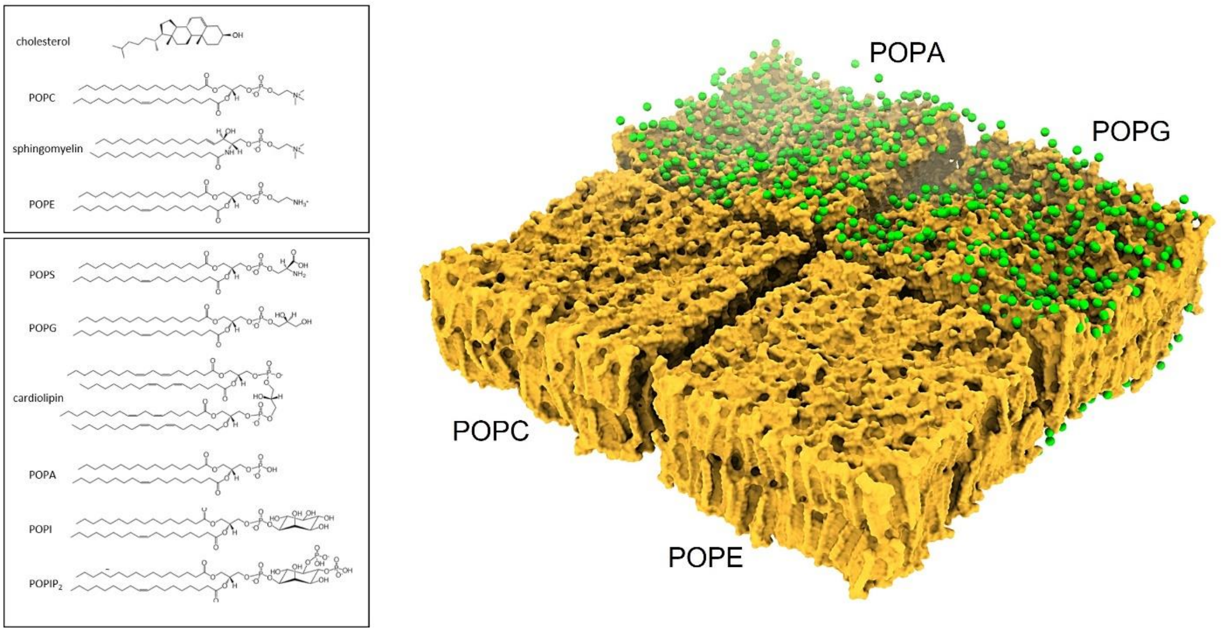


Figure S6. Left: chemical composition of the lipid molecules commonly found in mammalian (upper panel) and bacterial (lower) cell membranes. The head groups largely determine the membrane's surface configuration and govern its interactions with water, ions, and any approaching molecules. Right: molecular surface representations of the structures of lipid bilayers simulated in this study (snapshots after equilibration; potassium ions tightly bound to the anionic head groups shown in green)
